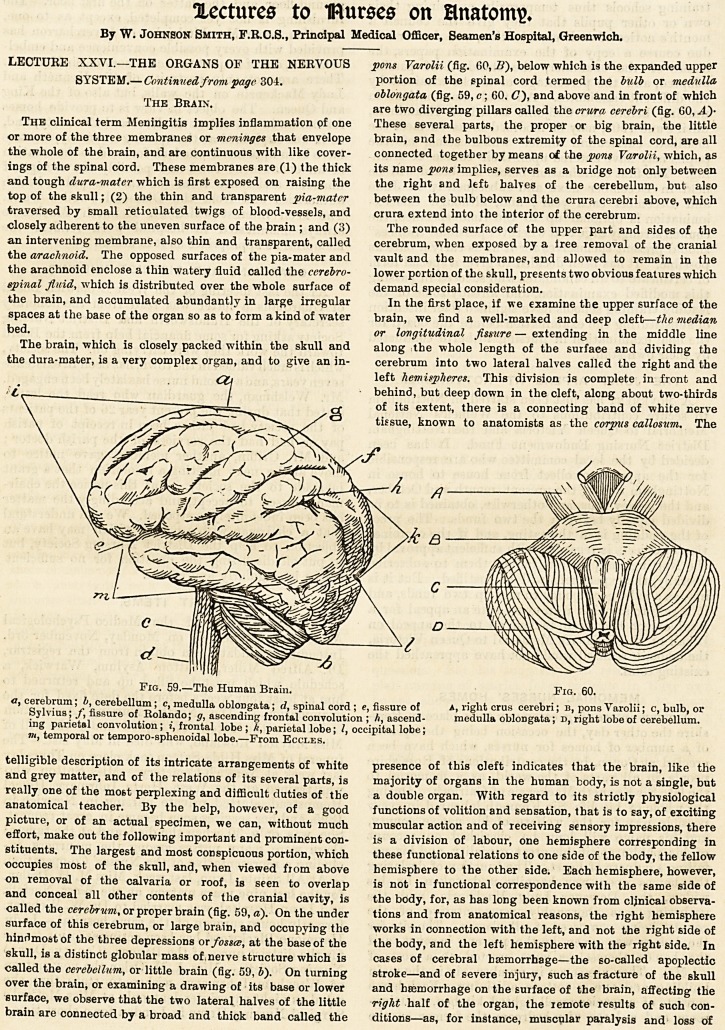# The Hospital. Nursing Section

**Published:** 1902-09-20

**Authors:** 


### The Hospital.

### Nursing Section.

Contributions for this Section of "THE HOSPITAL" should be addressed to the EDITOR, "THE HOSPITAL" NURSING SECTION, 28 & 29 Southampton Street, Strand, London, W.C.

No. 834.—VOL. XXXII. *SATURDAY, SEPTEMBER* 20, 1902.

### Notes on News from the Nursing World.

### MENTIONED IN A DESPATCH.

THE *London Gazette* last Friday contained, in addition to a list of honours bestowed by the King in recognition of the services rendered in the Lango and the Aro operations, a despatch from Sir R. Moor, High Commissioner of Southern Nigeria. This dispatch is dated Old Calabar, April 17th, and includes the report of Colonel Montanaro on the Aro expedition. Among the persons whom the commanding officer commends to the notice of the Commissioner is Nurse Graham. That Miss Graham had ample opportunities for manifesting her skill and devotion as a nurse may be gathered from the statistics given of the casualties. Thirteen Europeans were wounded; in addition to 27 native soldiers and carriers killed 150 were wounded, while 70 died of disease.

### QUEEN ALEXANDRA'S ROYAL NAVAL NURSING SERVICE.

THE changes which were contemplated in Queen Alexandra's Royal Naval Nursing Service when an account of an interview with the head sister at Haslar Hospital appeared in our columns, are now officially announced. The salaries of head sisters at Haslar, Plymouth, and Chatham are now to be identical, namely £125, rising by annual increments of £5 to £160. Nursing sisters, whose salaries formerly started at £30 rising to £50 by annual increments of £2, henceforth start at £37 and rise by instalments of £2 10s. to £50. Each head sister and nursing sister is to be allowed, in lieu of provisions and for washing, 15s. per week if connected with Haslar, Plymouth, or Chatham Hospital, 21s. if belonging to Malta, and 19s. if belonging to Dartmouth. Charge pay to the senior sister of a hospital ship is now 1s. 6d. a day. The period of leave for head sisters during the year is increased from 40 to 42 days, and that of nursing sisters from 32 to 35 days.

### AN AUSTRIAN DECORATION FOR A CHARING CROSS NURSE.

THE remarkable work done by Nurse Bourgignon, R.R.C., at Peking and Tientsin, has, we are pleased to learn, received further acknowledgment and honour from the Emperor of Austria. Miss Bourgignon has been presented with the decoration of the "Elizabeth" medal; an order which was established to commemorate the virtues and womanly qualities of the late Empress Elizabeth. The following extract is from the letter written by His Majesty's Secretary of Legation, in Peking, to Miss Bourgignon: "It affords me great pleasure to hand you herewith the Elizabeth Medal, which His Majesty the Emperor and King has been graciously pleased to confer on you for distinguished services in connection with the Queen Victoria Diamond Jubilee Hospital, during the China campaign in 1900." The decoration was presented to Miss Bourgignon along with baskets of flowers from the Consul and military officers of the dual empire; the gentlemen afterwards calling to express their congratulations personally, and also to tender their gratitude to her for her many kindnesses to their comrades. Devoted and unselfish work such as Miss Bourgignon's is worthy of recognition, and it must afford gratification to those in authority at Charing Cross Hospital to know that a member of that training school has, by her efficient and conscientious work, earned the admiration and gratitude of a great number of people in a far-off land.

### NURSES FROM SOUTH AFRICA.

THE following nurses arrived at Southampton on board the *Orotava*, Sisters E. M. Kent, E. Ambrose, and V. Christian (Civil); on board the *Soudan*, Sisters C. B. Drury, M. C. S. Knox, L. Allman, P. Schor, M. Potts, L. Cowley, A. McJ. Bower, G. W. Johnston, McAdam, S. Lamming, and K. A. Moxon; on the *Palawan*, the *Oriana*, and the *Orcana*, Sisters K. Parminter, S. E. Richmond, L. C. Noble, A. B. Buscoe, E. J. Wood, J. E. Dods, S. G. French, M. S. Milne, and E. Seddon Smith; on the *Walmer Castle*, Sisters C. H. Harrison and A. M. Thomson; on the *Golconda*, Sisters M. C. Cole, A. R. Alloway, L. Shepherd, E. J. M. Keene, M. Perceval, K. O. White, G. Black, S. F. Pollard, F. C. Puddicombe, M. D. Knapp, M. Clements, S. M. Lippiatt, M. Everett, A. Hill, and E. A. Cowley; on the *Ortana*, Sisters A. Fincher, M. J. Johnson, S. W. J. Hodden; and on the *Hawarden Castle*, Sisters M. McLeish, R. Howard, and A. Cook.

### THE NURSE CORPS OF THE AMERICAN ARMY.

AT the eleventh annual meeting of the Association of Military Surgeons of the United States, held in Washington, Dr. Anita Newcomb McGee, who organised the body of female nurses during the Spanish war, gave a short account of the development of the Army Nurse Corps. She advised the employment of female nurses as instructors of men belonging to the hospital corps in that branch of their duty, recommended post-graduate work in a military hospital for regular graduate nurses, and urged the establishment of a teaching force of a hundred nurses appointed for life and stationed for service at certain large hospitals, where they might also be available for detached service if needed. Dr. MeGee also expressed herself in favour of making provision for a war reserve, to be gradually created by admitting trained female nurses to any hospital in a constant succession of squads for a military post-graduate course, each nurse upon finishing the course to become a permanent member of the reserve until the suggested number of two thousand was reached. One of the officers who spoke at the conference alluded to the increased demand for female nurses in the British Army, and referred warmly to the value of their services. Another, while advocating the employment of women in large hospitals, considered their presence inadvisable at post hospitals, and impossible at ambulance stations and on the firing line. A third described the female nurse as "an indispensable feature of military hospitals," and expressed his belief that during peace they should be kept in constant training to serve as a nucleus of instruction and direction for the greatly expanded corps which he anticipates will be required upon the declaration of war.

### ALLEGED SOCIAL BOYCOTT BY NURSES.

The account of a week in a Nurses' Holiday Home, which appears in another column, is not pleasant reading. We may state that our correspondent has supplied us with the name of the home, and that the matron of the hospital with which it is supposed to be connected disavows any responsibility in the matter. But there is not, in fact, any complaint on the part of our correspondent against the management of the home, which seems, so far as the general arrangements and diet are concerned, to be admirably conducted. It is the attitude of the inmates against which, we think, she reasonably protests, seeing that she states that the manner in which she was treated by them is free from exaggeration. We can quite understand that a number of nurses away for a holiday, all friends together, were sorry when a stranger appeared in the home. But it was their obvious duty to dissemble their regret, and to show courtesy to the newcomer, who was no more an intruder than any one of themselves. It appears to us that in such circumstances there is only one course for the superintendent to pursue—namely, to plainly tell the nurses of the hospital with which it is associated that the expenses of the establishment are not covered by their payments, and that if they practically boycott outsiders, whether nurses or otherwise, they must either increase their weekly contributions, or face the alternative of the home being closed.

### THE BISHOP OF NORWICH AND THE NURSING MOVEMENT.

There has been opened at Norwich a new home in St. Martin-at-Palace Plain, which formally marks the fresh departure by the committee of the Norfolk and Norwich Staff of Hospital-trained Nurses mentioned by us on July 26th. The home is intended to supply nurses exclusively for the poor and the small tradespeople. There are six nurses attached to it, with Miss Creighton as sister-in-charge. They all sleep and live at the home, which has also accommodation for two paying patients. The Bishop of the diocese, who gave a short address at the service in St. Martin-at-Palace Church, which preceded the opening ceremony, said that the subscription would be something like a shilling a year, and that if at any time a poor person desired aid, and he was not a subscriber, then a nurse would be sent if he agreed to pay the contribution in future. "The work of the Norfolk and Norwich staff of nurses," continued the Bishop, "is greatly appreciated, and there is an increasing demand from the poor for trained service." He warmly appealed for subscriptions to carry on "so useful and beneficent a work."

### THE BETTER NURSING OF THE INSANE.

In our issue to-day Dr. Louis Vintras puts in a strong plea for the better nursing of the insane. He maintains that the salaries given to attendants are inadequate, and that no inducement is offered to encourage them to devote themselves to an exacting, "and at first sight, not over attractive speciality." On the question of special training he has much that is forcible to say. He rightly emphasises, for example, the need of training being not purely practical but to a large extent psychological. It is, as he asserts, the understanding of the characteristics of the mentally afflicted which is of such paramount importance if their confidence and obedience are to be gained. His ambition is to elevate asylums to the level of hospitals, and, in fact, to have them regarded in that light. There is a vast deal to be done, so far as nursing is concerned, before this ideal can be reached.

### HOME NURSING VERSUS HOSPITAL TREATMENT.

At the inquiry which has been held in York by Dr. R. W. Johnstone, the inspector of the Local Government Board, into the application of the City Council for power to borrow money for the purchase of land to extend the Fever Hospital, Mr. A. J. E. Parker declared that it was much better to leave scarlet fever cases in their own homes than to take them through the crowded streets, to congregate them all together in one large hospital. He added that it was his belief "that the patients would be better nursed at home." The inspector concluded that Mr. Parker was not an expert with medical training; but the latter gave a list of his qualifications, and, as he was Medical Officer of Health for the southern district of Houghton-le-Spring for 20 years, his professional position is beyond question. But his suggestion to resort to the old-plan nursing of scarlet fever patients in their own homes shows how widely men differ as to many points which have long been regarded as definitely decided.

### THE TRAINING OF NURSES IN VICTORIA.

From "Beyond the Seas" we have received a copy of a circular letter issued by the council of the Victorian Trained Nurses' Association to registered training schools for nurses. The council, we learn, has now recognised 35 metropolitan and country hospitals in the colony as general training schools, and the Women's Hospital at Melbourne for training in midwifery and gynæcology. On and after June 1905 no final examination in nursing will be recognised by the council as qualifying for registration by the Association except those that are to be held twice a year by its duly constituted conjoint Board of Education. Up to that date "trainees" from recognised training schools with not fewer than 40 beds may present themselves for their final examination in nursing in their own, or in any other, registered training school. It is required, however, of all training schools thus temporarily examining their own or other pupils that they give the council a month's notice of each examination, and forward in due course a copy of the examination papers, the names of the examiner, the standard of passing adopted, and the percentage of marks attained by the different candidates. All final examinations in nursing before the Conjoint Board of Examiners, until June 1905, may be modified so as to meet the modified curriculum under which some of the pupils may have entered upon their course of training. The first examination before the board will be held in December next and subsequently in June and December each year; and, although this examination is optional up to the time specified, the council strongly urge that wherever possible "trainees" should present themselves for it at as early a date as possible. It has been decided to give a special form of certificate to all candidates who succeed in passing this modified examination, and already some of the leading training schools have signified their intention of substituting the examination before the board for the final examination in nursing hitherto conducted by their own staff.

### THE JOINT APPEAL AT NOTTINGHAM.

A movement has just been started in Nottingham to secure contributions to the Women's National Memorial to Queen Victoria and the Nottingham District Nursing Endowment Fund. It has been decided by the local committee who are responsible for the appeal to collect from house to house in Nottingham during the present month and October, and the money thus, and otherwise, obtained is to be divided equally between the two funds. The proof of the pudding is in the eating, and if the combination of objects is regarded with sufficient approval by the Nottingham people to induce them to subscribe liberally, the experiment will be justified. But it is not, as a rule, good policy to mix up two funds, and it is safe to say that if in every case an appeal for a local object had been tacked on to the appeal on behalf of the Women's Memorial to Queen Victoria, the amount realised would not have approached the existing total.

### MEMORIAL NURSES' HOMES.

A very interesting ceremony took place in Ross-shire the other day, the occasion being the opening of a number of homes for nurses, which have been erected at Conon, by the contributions of Ross-shire people in all parts of the world, as a memorial to the late Sir Kenneth Mackenzie of Gairloch. During the whole of his life Sir Kenneth laboured in every possible way for the welfare of his native county, and his name will long be cherished on account of his public spirit, his great geniality, and his unfailing kindness of heart. The function of declaring the homes open was happily undertaken by his widow, the Dowager Lady Mackenzie, and the gathering was thoroughly representative of the county, being composed of all classes. The site for the homes was given by the present baronet, and the homes themselves were erected at a cost of £1,500. They are seven in number, six in the main building and one a cottage adjoining. Each home consists of a spacious well-lighted kitchen, a sitting-room, and a large airy bedroom, the former on the ground floor and the latter on the first floor. The furnishing is not yet completed, except as to one, and this home Mr. Littlejohn of Invercharron has provided with every possible convenience and embellishment, out of respect to the memory of Sir Kenneth. There are not only portraits of Sir Kenneth and Lady Mackenzie on the walls, but also of the King and Queen. The object in view is to provide homes for nurses in the shires of Ross and Cromarty, and, as it was explained at the opening ceremony, trustees have been appointed who look forward to the creation of an endowment fund and possibly the receipt of financial assistance from the various public-health authorities and from the Diamond Jubilee Fund. The keen interest which Sir Kenneth Mackenzie always manifested in hospital work renders the memorial as appropriate as it is useful.

### GUARDIANS AND DISTRICT NURSING.

At the last meeting of the West Bromwich Board of Guardians a letter was read from the honorary secretary of the Handsworth and District Nursing Society asking for some financial help from the Board toward the work they were carrying on. The society, which is much valued in the town, has been in existence seven years, and a second nurse has lately been engaged. Mr. Welshman, the guardian who read the letter, stated that during the present year 26 of the patients of the society had either been in receipt of parish pay or had had the services of the parish doctor; and Mr. Griffin, another member, gave notice to move at the next meeting a resolution that a grant be made to the society. But this notice the chairman refused to accept, and accordingly the matter has been indefinitely postponed. We can understand that a chairman with a narrow vision may have an objection to help the District Nursing Society, but to put off discussing the question for no sufficient reason is singularly ungracious.

### SHORT ITEMS.

The examination of the Medico-Psychological Association will be held on Monday, November 3rd. Intending candidates can obtain from the registrar, Dr. Alfred Miller, Hatton Asylum, Warwick, a schedule which must be filled up and returned to him at least four weeks before the date fixed for the examination. — The Neath Nursing Association benefits to the extent of £2,000 under the will of Miss Mayzod Rowland, who died in May last.—The funeral of Miss Hilda Sutton took place on Thursday last week at Norwich Cemetery. Miss Sutton, who was trained at the West Herts Infirmary, Hemel Hempstead, started private nursing in Norwich in 1895, and her services soon became in great request. She was only 30 when she died, and at the funeral there were many nurses among the mourners, including a representative of the Nurses' Co-operation.—At All Saints, Lynn, last week, Miss M. H. Dalley, for the last nine years matron at the West Norfolk and Lynn Hospital, was married to Dr. W. Webster, formerly house surgeon of the same hospital.—Lady Hermione Blackwood has been acting as *locum tenens* for the district nurse of the Bangor (County Down) Nursing Society.—Miss A. M. Oslar, the new matron of Wrexham Infirmary, wishes to state that she acted as night superintendent only at Miss McCaul's Surgical Nursing Home in London for a short time.

### Lectures to Nurses on Anatomy.

By W. JOHNSON SMITH, F.R.C.S., Principal Medical Officer, Seamen's Hospital, Greenwich.

### LECTURE XXVI.—THE ORGANS OF THE NERVOUS SYSTEM.—*Continued from page* 304.

### THE BRAIN.

THE clinical term Meningitis implies inflammation of one or more of the three membranes or *meninges* that envelope the whole of the brain, and are continuous with like coverings of the spinal cord. These membranes are (1) the thick and tough *dura-mater* which is first exposed on raising the top of the skull; (2) the thin and transparent *pia-mater* traversed by small reticulated twigs of blood-vessels, and closely adherent to the uneven surface of the brain; and (3) an intervening membrane, also thin and transparent, called the *arachnoid.* The opposed surfaces of the pia-mater and the arachnoid enclose a thin watery fluid called the *cerebro-spinal fluid*, which is distributed over the whole surface of the brain, and accumulated abundantly in large irregular spaces at the base of the organ so as to form a kind of water bed.

The brain, which is closely packed within the skull and the dura-mater, is a very complex organ, and to give an intelligible description of its intricate arrangements of white and grey matter, and of the relations of its several parts, is really one of the most perplexing and difficult duties of the anatomical teacher. By the help, however, of a good picture, or of an actual specimen, we can, without much effort, make out the following important and prominent constituents. The largest and most conspicuous portion, which occupies most of the skull, and, when viewed from above on removal of the calvaria or roof, is seen to overlap and conceal all other contents of the cranial cavity, is called the *cerebrum*, or proper brain (fig. 59, *a*). On the under surface of this cerebrum, or large brain, and occupying the hindmost of the three depressions or *fossæ*, at the base of the skull, is a distinct globular mass of nerve structure which is called the *cerebellum*, or little brain (fig. 59, *b*). On turning over the brain, or examining a drawing of its base or lower surface, we observe that the two lateral halves of the little brain are connected by a broad and thick band called the *pons Varolii* (fig. 60, *B*), below which is the expanded upper portion of the spinal cord termed the *bulb* or *medulla oblongata* (fig. 59, *c*; 60, *C*), and above and in front of which are two diverging pillars called the *crura cerebri* (fig. 60, *A*). These several parts, the proper or big brain, the little brain, and the bulbous extremity of the spinal cord, are all connected together by means of the *pons Varolii*, which, as its name *pons* implies, serves as a bridge not only between the right and left halves of the cerebellum, but also between the bulb below and the crura cerebri above, which crura extend into the interior of the cerebrum.

The rounded surface of the upper part and sides of the cerebrum, when exposed by a free removal of the cranial vault and the membranes, and allowed to remain in the lower portion of the skull, presents two obvious features which demand special consideration.

In the first place, if we examine the upper surface of the brain, we find a well-marked and deep cleft—*the median or longitudinal fissure*— extending in the middle line along the whole length of the surface and dividing the cerebrum into two lateral halves called the right and the left *hemispheres.* This division is complete in front and behind, but deep down in the cleft, along about two-thirds of its extent, there is a connecting band of white nerve tissue, known to anatomists as the *corpus callosum.* The presence of this cleft indicates that the brain, like the majority of organs in the human body, is not a single, but a double organ. With regard to its strictly physiological functions of volition and sensation, that is to say, of exciting muscular action and of receiving sensory impressions, there is a division of labour, one hemisphere corresponding in these functional relations to one side of the body, the fellow hemisphere to the other side. Each hemisphere, however, is not in functional correspondence with the same side of the body, for, as has long been known from clinical observations and from anatomical reasons, the right hemisphere works in connection with the left, and not the right side of the body, and the left hemisphere with the right side. In cases of cerebral hæmorrhage—the so-called apoplectic stroke—and of severe injury, such as fracture of the skull and hæmorrhage on the surface of the brain, affecting the *right* half of the organ, the remote results of such conditions—as, for instance, muscular paralysis and loss of

FIG. 59.—The Human Brain.

*a*, cerebrum; *b*, cerebellum; *c*, medulla oblongata; *d*, spinal cord; *e*, fissure of Sylvius; *f*, fissure of Rolando; *g*, ascending frontal convolution; *h*, ascending parietal convolution; *i*, frontal lobe; *k*, parietal lobe; *l*, occipital lobe; *m*, temporal or temporo-sphenoidal lobe.—From ECCLES.

FIG. 60.

A, right crus cerebri; B, pons Varolii; C, bulb, or medulla oblongata; D, right lobe of cerebellum.

LECTURES TO NURSES ON ANATOMY.—*Continued.*

sensation—will be found on the opposite and *left*, half of the body. This physiological fact is so constant, and so well established, that, in any case of paralysis or of convulsions affecting one side only of the body, we may confidently declare that the primary cause of such mischief exists in the opposite side of the brain. To this rule that each half of the body is thus associated with the opposite half of the brain there is a remarkable exception in the fact that the nerve centre—*Broca's convolution*—controlling the function of speech is situated on one side only of the brain—on the left side with the large majority who are right-handed, on the right side with the few who make more use of the left hand.

The surface of the brain, which in most animals is quite smooth, presents in man a very complicated pattern of folds and intermediate depressions. By this arrangement a large extent of grey matter which is spread over the surfaces of the hemispheres is packed within the narrow and unyielding limits of the skull. The sinuous folds are called *convolutions* and the depressions *fissures or sulci*. In the course of the last quarter of a century anatomists have succeeded in forming a reliable map, by means of which what had previously been regarded as a bewildering and capricious arrangement of folds and furrows may now in almost every human brain be clearly comprehended as a constant grouping of well-defined regions. The principal guides in the topographical study of the surface of the brain are (1) a very deep and irregular fissure, called the fissure of Sylvius (fig. 59, *e*), which, starting from the under surface of the brain at the junction of the anterior and middle thirds of this surface, passes at first upwards and afterwards almost directly backwards along the outer surface, sending off in its courses several small branches; and (2) the fissure of Rolando (fig. 59), which runs downwards and forwards along the outer surface of the hemisphere from a point corresponding to about the middle of its upper margin. The external surface of each hemisphere has been mapped out into four lobes, each of which contains three or four large and contorted convolutions, taking its name from the cranial bone to which it corresponds. Thus the portion of brain surface in front of the fissure of Rolando is called the *frontal lobe* (fig. 59, *i*), that behind the fissure the *parietal lobe* (fig. 59, *k*), which lobe is separated by a well-marked depression called the parieto-occipital fissure from three other convolutions placed quite at the back of the brain, which constitute the occipital lobe (fig. 59, *l*). The three well-marked convolutions which run below and parallel to the fissure of Sylvius form the *temporal*, or, as it is also called, the *temporo-sphenoidal lobe* (fig. 59, *m*).

The last-mentioned lobe is the usual situation of the variety of abscess of the brain that is so often due to chronic and neglected discharge from the ear.

Before we complete this brief and perhaps not very interesting review of the surface topography of the brain, we must not neglect to direct special attention to two well-marked convolutions which form, one the anterior, the other the posterior, boundary of the fissure of Rolando. The former of these, forming the posterior part of the frontal lobe, is called the *ascending frontal convolution*, and the latter, forming the front portion of the parietal lobe, is called the *ascending parietal convolution* (fig. 59, *g* and *h*). These two convolutions are the seats of the *motor areas* of the brain, in which, as has of late been proved by physiological research and by clinical observation in cases of cerebral injury and disease, are seated the centres or primary sources of the force by which muscular movements are effected on the opposite side of the body. The different centres, which are quite distinct though closely packed together, present a somewhat complex arrangement, but we may form a general idea of their disposition by remembering that they are placed in a reversed order to that of the parts of the body to which they correspond, those for the lower limb being in the upper part those for the upper limb in the lower part of each convolution, and those for the head, neck, and face being in the middle. These centres, we must bear in mind, are not distinguishable by any apparent differences in structure or colour, the parts included in the motor area being quite uniform and not presenting, even on microscopical examination, any structural divergence from other parts of the surfaces of the cerebral hemispheres.

### Nursing at the Royal Portsmouth Hospital.

BY OUR COMMISSIONER.

FOR some time past the affairs of the Royal Portsmouth Hospital have been prominent, and as the result of much agitation there has lately been a considerable infusion of new blood into the committee. It may be hoped that better management and time will bring about the restoration of public confidence; for until this is gained there will inevitably be a slackness in the response to the appeal which is now being put forward for further much-needed funds. In visiting Portsmouth the other day, however, it was not my object to investigate the charges of a general character which have formed the subject of a somewhat embittered controversy in the local papers, but, in view of the appointment of the new matron, to ascertain the nature of the nursing arrangements. For these, of course, Miss Atkins is in no way responsible. In fact, she had only entered upon her duties the evening prior to my visit, and though she showed me round the wards, and gave me all the information she could, I did not, in the circumstances, ask her to express an opinion respecting any of the rules now in force.

### THE WARDS AND THE NURSES' QUARTERS.

As all who know the building in Fitzherbert Street are aware, the Royal Portsmouth Hospital is pleasantly situated, and has no lack of ground for purposes of outdoor exercise and recreation. The number of new wards is four, and each contains 21 beds. They are light, airy, and well-equipped; but seeing that they were only erected in 1898, it is not satisfactory for the floors to crack. The children's ward contains 36 beds, and there are beds in the old wards for 30 lock patients, making in all 150. In a separate building, called "the Hut," there are four beds for isolation cases, and here is an up-to-date theatre, an out-patients' department, with a large dispensary, and kitchens of sufficient dimensions. In going over the hospital it was impossible not to be struck by the amount of cleaning which is rendered imperative owing to the many corridors and passages that have to be traversed. This must help to increase the labour of the nurses. The want

### NURSING AT THE ROYAL PORTSMOUTH HOSPITAL.—*Continued.*

of a nurses' home is also obvious. Each nurse has a bed-room of her own, which is heated by hot water; there is a general dining-room of fair size; and the sisters and nurses have a sitting-room each—with a ping-pong subscribed to by the sisters themselves in the sisters' room, and a piano in that of the nurses. But the accommodation is not all that could be desired. The two nurses in attendance on the lock patients have a room attached to each ward. No probationers are employed in these wards.

### The Sisters.

With regard to the work of the nursing staff, there are five sisters—a sister to each ward—on duty during the day, the home sister, who is also assistant matron, and a night sister. The number of probationers is 28; those in their third year of training are called staff nurses. Special rules for the sisters, which, together with those for the probationers, were drawn up under the auspices of the late matron two years ago, do not call for comment. Naturally as probationers are admitted at the age of 22, a fully-trained nurse may become a sister at the age of 25. The hours of of duty and salaries are not stated in the regulations; but the former are from 8 A.M. to 8.45 P.M., and the latter is £30 per annum. The sisters have four weeks' holiday, a day monthly and two hours daily off duty.

### The Probationers.

There are two classes of probationers. Some are received for training for one year, in which case they are required to pay a fee of thirty guineas to the hospital. At the end of the year they may, with the consent of the house committee upon the recommendation of the matron, continue their training for another two years on the same terms as the ordinary probationers. A certificate is only given at the end of three years. Every probationer pays a guinea prior to her month of trial. At the end of the month, if satisfaction has been given to the matron, she is interviewed by the house committee, with whom she is required to enter into an agreement to remain in the service of the hospital for three years from the day of joining. She is then provided with three print dresses, aprons, caps, sleeves, and collars. These are issued annually by the matron, but each probationer has to provide, at her own expense, a cloak and bonnet of a uniform pattern approved by the matron. Outdoor uniform is thus, under the existing rules, obligatory. If a probationer wishes to leave before the expiration of her term without the consent of the house committee, she forfeits two months' salary as well as her certificate. No probationer is employed on night duty during the first six months of her service, nor at any time for more than six months consecutively. Here it may be interpolated that there are few probationers who can stand the strain of six months on night duty.

### A "Crown of Merit."

Probationers are on duty from 7 A.M. to 8 P.M.; they have three weeks' holiday, a day monthly and two hours once a week off duty; and their salary is £8 the first year, £14 the second, and £20 the third year. On the recommendation of the matron, a probationer may receive "a crown of merit after two years' approved service," entitling her to an extra £2 a year. Indeed, she is entitled to exact it. The nurses whose services fail to be satisfactory to the matron go without it. It will strike a good many people that the institution of the "crown of merit" has drawbacks, and may easily be converted "into a sort of preliminary test." Courses of lectures are given by the honorary staff to the nurses each year and there is an examination after each series, but no final examination.

### The Certificate.

The certificate is worded as follows:—"This is to certify that ——— has been trained for the full term of three years in the medical, surgical, and children's wards of this Hospital, has received instruction in the art of nursing, and that she is now competent to undertake the duties of a trained nurse." Signed by the chairman, the two surgeons, and the matron, it occupies a very small space, and there is absolutely no room on the paper for remarks of any kind. Thus, the certificate not only states that a nurse has received her three years' training at the Royal Portsmouth Hospital, but also that she is "competent to perform the duties of a trained nurse." Clearly such a certificate should not be lightly signed, but when signed any system which exposes the nurse to the risk of having the value of her certificate destroyed by supplementary information furnished behind her back is one to deprecate. If a nurse does not merit a certificate she ought not to have it given her; and if she does, there should be no possibility of it proving a useless possession so long as her conduct remains satisfactory.

### Private Nurses.

There is a staff of eight private nurses attached to the Royal Portsmouth Hospital, who commence with a salary of £32, rising £2 per annum to £36 a year, with board, lodging, washing, and indoor uniform. As a general rule, I gather that they are all occupied, and it is proposed to increase the number to twelve. They are under the control of the matron, are given three weeks' holiday in the year, and each nurse is allowed, "if possible," one day's holiday in each case after her return from nursing. They are required to "hold sacred any knowledge they may obtain of the private affairs of the family in which they are engaged, and to adapt themselves as far as possible to the usages of the family"—which is all very desirable, but, unless the matron makes private inquiries, one wonders how it can be ascertained whether the stipulation is faithfully carried into effect.

### A Week in a Nurses' Holiday Home.

BY A TRAINED NURSE.

I am generally considered a harmless person, somewhat lively, but good natured withal, and look on most things with an optimistic eye, but I have received a severe shock, and I scarcely know whether I am a nurse or not now. True I have a three years' certificate from one of the biggest London hospitals, also my L.O.S. certificate, but during the past week I have heard so often—directly and indirectly—that I am not a nurse, that, like the Scotchman, I hae ma doots. For the first time in my nursing career of twelve years I have felt ashamed of my companions. My tale will speak for itself. I am a married woman, but as my husband's work takes him abroad a good deal, when I cannot accompany him I fall back on my profession to keep me occupied, and therefore happy, till he returns.

### A Pleasant Change.

This time I had some difficulty in getting work, so I thought that it would be a pleasant change to spend a few weeks in a nursing home; here I believed I should find congenial companions, and pass the time pleasantly till my

### A WEEK IN A NURSES' HOLIDAY HOME.—*Continued.*

husband returned. I selected one, and wrote to the superintendent to ask if she could take me in. She answered in the affirmative, so I went down the next day. But I was entirely ignorant of the fact that the nurses who were in the habit of using the home considered that admission to it was their exclusive right. I found the house charming. Beautiful lawns, plenty of flowers, comfortable chairs, and no irksome rules; punctuality at meals was required, and we were requested to make our own beds and keep our rooms tidy. A neatly dressed maid ushered me in. While I waited in the hall a nurse rushed across into an adjoining room, exclaiming in a loud voice, with uplifted hands, that a lady had arrived with a box as big as Noah's Ark. It was only an ordinary ladies' dress-basket, and as there was plenty of room for it in my bedroom, and a porter to carry it upstairs, I do not think the size mattered much to anyone but myself. However, the remark made me uncomfortable, so not wishing anyone to think I had entered the Home under false pretences, I asked to see the superintendent, and explained to her that if possible I would like to remain three or four weeks, but she was on no account to turn away a nurse for want of room while I was there, as I was quite willing to give mine up if necessary, being able to pay for apartments elsewhere, which many a poor nurse could not do. She assured me there would be no necessity for that, and that they frequently took in outsiders—by "outsiders" I suppose she meant people unconnected with the nursing profession. I heard afterwards that the Home did not pay its way, and the committee of the hospital who ran it were anxious to make it better known, hence the outsiders.

### Nine Pairs of Eyes.

Having cleared my conscience I went downstairs. Sounds of laughter and conversation directed me to the drawing-room, so opening the door I walked into—dead silence. The talking had stopped as suddenly as if the inmates were wound-up automata, and the mainspring had given way. No one offered me a greeting, so, feeling as if a bucket of cold water had been thrown over me, I walked to a seat, and picking up a newspaper which providentially lay near at hand, shielded myself from the glare of nine pairs of eyes. The oppressive silence was broken by the clanging of a bell, when everyone rose and filed silently into the dining-room. Here, I hoped, under the influence of a good supper they might thaw. I made heroic but fruitless attempts to start a conversation, so after complying with several requests to pass the butter, and hand the mustard, I succumbed to the "wet blanket" treatment. I retired early that night.

### The Heights of Frigid Nursedom.

The next morning's breakfast was a repetition of supper. I am sure I won my V.C. over and over again storming the inaccessible heights of frigid nursedom. Curt monosyllabic replies to a question that demanded an answer, and if I hazarded a general remark it was received in frozen silence. After breakfast everyone stood up, grace was said by the superintendent, and I walked to the door opening on to the lawn, intending to escape that way, when a confused murmur of voices arrested my footsteps. Turning round, to my astonishment I saw that they were all at prayers. No one had had the courtesy to warn me of it beforehand. On the contrary my embarrassment seemed to afford them amusement. After prayers we all separated and went our own ways till dinner at 1.30. This was a repetition of the other meals. The funny thing about it to me was, that all the nurses seemed cut out of the same pattern, like a flock of sheep. I do not think I ever met such wholesale lack of individuality, and it puzzled me very much, till I learnt later on, that they all belonged to the same hospital, and resented the introduction of any other element, regarding it as an intrusion into their own sacred domain. If it be true that the Home cannot pay its way, it seems to me, to use a homely simile, that the nurses were "cutting off their noses to spite their faces."

### The Wit of the Party.

I was there a week. Nurses came, and nurses went, and with hardly an exception they all behaved in the same odious manner. Their conversation, the audible part of it, was mostly personal or reminiscent; the rest was carried on in giggling whispers with an occasional loud burst of laughter. Happening to raise my eyes from my book one day to see what the unusually loud laughter was about, I found the "wit" of the party busily engaged in taking me off; and yet this nurse had accepted some delicious French sweets from me that very afternoon, which I had offered with a forlorn hope of making her friendly. I was especially careful that the contents of my "Noah's Ark" should not give offence, so I limited myself to a serge skirt and flannel or cotton blouses. I might have spared myself the trouble, as there was quite an eruption of silk and satin blouses, and elaborately trimmed costumes with sweeping trains, so doubtless I was not the only envied possessor of an "ark."

### Another Victim.

I was not the only victim of the social boycott. Perhaps being independent, I knew I could put a stop to it by leaving whenever I wished to do so, but there was an unfortunate girl—she was a nurse, too—who suffered the agonies which only a delicate, sensitive mind can suffer. Her entrance into a room was invariably the signal for all the rest to leave it, no matter how they were employed. I have heard her mild request for the bread and butter to be passed her at tea quite ignored, and have jumped up and handed it to her myself; in fact they made life so unbearable for her that she left two days after I did.

### A Personal Affront.

The climax came one afternoon. I had gone out intending to call on a friend, and had left word that I should not be in for tea. My friend was out, so I hurried back hoping to be in time. They had barely finished, so I asked the presiding genius of the teapot to give me a cup, as I was very hot and thirsty. She handed me some diluted hot water which I meekly swallowed, and then *apropos* of nothing at all she burst out furiously that the Home was supported by the hard-won earnings of the private staff of the hospital, and that it was a shame that people who were not nurses should take advantage of it to the detriment of the nurses themselves. The remark was aimed at me, so I replied that I was a nurse; but I could not quite agree with her opinions as I presumed the Home was run on business lines, and the committee knew what they were about. Having paid in advance I was determined to see the thing through, but the superintendent observing how unpleasant things were asked me to give up my room to a nurse who was expected—a polite fiction to which I gratefully consented. The above is not in the slightest degree exaggerated. I honestly did my best to be cheerful and polite, and promote a little kindlier feeling. I obeyed all the rules and abstained most carefully from giving offence; but it was not the slightest use. If other nurses have been treated as I have been I am not surprised that the Home is unpopular; and it is a pity that the selfish behaviour of a few women should deprive others of a really comfortable Home when they need rest.

### Presentations.

Maternity Hospital, Glasgow.—The nursing staff and pupils of the Glasgow Maternity Hospital have presented Miss J. Rogers with a travelling clock suitably inscribed. She has been on the staff of the above hospital for over four years, and is leaving to take a matron's post.

### The Nursing of the Insane.

BY LOUIS VINTRAS, M.D., B.Sc.

Of all the branches of nursing that of the insane is perhaps the one that has received less recognition, and yet of all it is, in many respects, the most interesting. It requires a vocation to make a good nurse, it requires something more to make a good asylum nurse. Love of the work, *per se*, will not suffice in this case, it will require also a sense of humanitarianism. Unless the nurse has wide human sympathies, little indeed will be the influence that she can hope to exercise over those suffering from the scourge of insanity. Here she is no longer dealing with rational beings, adults or children, but with a strange mixture of both; beings who are mentally as children and physically men and women. In this is the secret of the difficulty, for whereas healthy-minded men and women and healthy-minded children are straightforward, though distinct problems to deal with, this unnatural combination of mental childhood with physical adultness is responsible for much of that which makes madness so often repulsive. None should embark on so delicate a calling without having first well weighed not only her personal inclination, but also her physical and mental aptitude for such work. Not that I share the current belief that only those who are strong of frame and matter of fact make good nurses for the insane; far from it. Those who form the majority of this type are generally too direct of mind, too decided of purpose to be able or willing to occupy themselves with fine and intricate distinctions; they are given to generalise, while in dealing with the insane one must always individualise. One of the causes of the little progress we have made in dealing with insanity in general is that we have divided it up into well defined categories and treated it accordingly; we have not considered enough the element of individuality. What I understand in this particular case by physical aptitude is a healthy tone and perfect control of the nervous system, and by mental aptitude a freedom from any tendency to dwell on the dark side of things in general, a disposition to look upon the accidents of life and of nature merely as the backward swing of the pendulum, by which it gains a new impetus to rise again on the other side.

### Higher Salaries Needed.

Nursing of the insane requires a long probation, a long and tedious training and this at present is in many cases most perfunctory. This must necessarily be so while the salaries offered remain inadequate. No inducement is held out to encourage nurses to devote themselves to this exacting and, at first sight, not over-attractive speciality. This branch of the nursing profession should be highly retributed, much more highly than any other, and this in proportion from the very beginning of the stage of probation. It should be sought to attract and retain those elements of the profession which are of the highest order, for in dealing with patients suffering from mental diseases, the personal equation of the attendant plays an even greater part than is the case in dealing with ordinary patients. Regular courses should be started, examinations held at the end of the first and second year, and certificates delivered to those who are successful; thus in time a staff of nurses truly competent in this branch of science would be available for every asylum, and a more rational treatment of insanity be thereby made more possible than it is now. If we would raise the general treatment of the insane from its present empiricism to a level more in accordance with the great humanitarian ideals of the age, we can only do so by first thoroughly training and organising all those who are to co-operate in the work; for however excellent the system, if its foundation be not solid, no good can be expected of its application.

### Special Training.

Now as to special training of nurses for asylum duty, it should be not only purely practical but to a large extent psychological. It is the understanding of the characteristics of the mentally afflicted which is of such paramount importance, if their confidence and obedience are to be gained, and as yet little or nothing is done in this direction. The insane must not be looked upon merely as broken and perverted machines, but as beings in whom certain original or accidental flaws are responsible for certain proclivities and in the management of whom these flaws must always be taken into intelligent consideration. It is this that will enable the nurse or the attendant to differentiate between mere wilfulness on the part of the patient and faults inherent to his diseased condition, and enable a just decision as to when firmness or indulgence will prove most beneficial. Again, in the ordinary duties of nursing, there are ways of approaching these patients which are different from those employed towards other sick people. Here routine alone is not to be depended upon, and the individual element in each case, will, if properly understood, give an interest to the most ordinary duties. Seen from such points of view as this, the nursing of the insane becomes an occupation with no small sense of attractiveness about it, because it presents infinite variety. The nurse must also have some knowledge of the ætiology of the various forms of mental disease, so that she may be able to prevent or counteract trifling incidents or remote suggestions acting on the patient's mind and dangerous as possibly capable of exciting the patient and leading up to paroxysms of violence. I am quite aware that in an asylum where many hundred insane are living together much must naturally remain imperfect, and that intention cannot at present be carried out to the full; but we should be always preparing ourselves to grapple more and more efficiently with the problem of insanity, until such time as we can look forward with more certainty to the application of curative methods.

### In the Light of Hospitals.

To this end we modern people must first of all give up looking on our asylums as permanent retreats for incurables, to consider them in the light of hospitals, and it is those who are in hourly contact with the patients who can do so much in this direction, if they start with the comprehension of the full possibilities of the work to which they are devoting themselves. For in this great question of the insane, there is more than the interest and satisfaction appertaining to all good work; there is a high humanitarian ideal underlying it, perhaps the highest ever attempted by man—the ultimate reclaiming of fellow creatures from the living darkness of their minds. Amid all the social problems it is one of the greatest, and has so far been one of the most neglected. Much sympathy, money, and endeavour has been lavished on the criminal, who, whatever the remote cause of his proclivities, preys on society for his own gain; but what are his claims to our pity or to our help compared with that of fellow creatures whom we are obliged to banish from our midst, and deprive of their liberty through no fault of their own.

### The Instinct of Alertness.

But the nurse in remembering her duties to the patients, must not be forgetful of those to herself, and the knowledge she has gained will enable her to recognise those among the

THE NURSING OF THE INSANE.—*Continued.*

inmates who are or may become dangerous, and to be on her guard in consequence. This knowledge will also give her confidence, and prevent her from being nervous. But at all times she will have to exercise that instinct which is born of habit—the instinct of alertness; for it can never be said of a person mentally afflicted that he will not at some time or another become violent, and only rapidity of action, in wards where many inmates live in common, will prevent such sudden and often unexpected outbursts from having serious consequences either to the patient himself or others. Again, she must, when off duty, be able to disassociate her thoughts entirely from her work, and find lively and congenial society or pastimes. In all asylums the nurses' quarters should be as isolated from the wards as possible, and wherever feasible in a separate house; their rooms should be made comfortable and attractive, and they should be afforded every facility for healthy outdoor exercise and for pleasant pastimes when in their quarters. I should strongly recommend all nurses in asylums to take up some hobby: photography, or anything of the sort, and facilities for such should be afforded them by the authorities.

THE MALE ATTENDANTS.

What I have said for the nurses applies, of course, to the male attendants, and the choice and training of the latter should be, if anything, even more carefully looked into. Men have not the same pliability as women, and soon take things as a matter of habit, and often become mere punctual machines when employed in institutions; against this those in authority must always be on their guard and find some means of interesting and encouraging the men in their work. The fault is, in this case as in that of the nurses, that the salaries are not sufficiently high and that here, moreover, there is no, or little, advancement to look forward to.

### Some Ideas about Holidays.

BY A SISTER.

SUCH a dismal moment it is when the holidays are over! Everything pleasant past for the time, and work, work, work staring us in the face, and threatening to abide with us for the next eleven months. Not that we object to our work; it is the dearest thing in the world to some of us; but we dislike that moment when we are scarcely divided from our delightful month of irresponsibility and "do-as-you-like," and yet have made our plunge into the sea of work. It is almost too much for us; we have lost our breath, cannot see quite plainly, we think the next wave coming will be too strong for us, and we wish ourselves back on the shore of repose. It was such a pleasant holiday! Our friends laid themselves out to give us a good time, and if the rain did rain nearly every day, it soon dried up again after washing the dusty hedges; and the fields were not so brown as last summer, nor was it so impossible to cycle or walk in the middle of the day.

Holidays are a great invention, but it takes a wise woman to lay them out so as to get every possible benefit from them. Perhaps the chief thing to be sought after in a holiday is that kind of rest which consists in change of work. One does not imagine that the ideal holiday for a postman would be a walking tour in his own county, though it might be the most picturesque of all the shires of England. Nor does an expedition on foot recommend itself to all nurses, in spite of its being to many minds the most fascinating way of seeing the world. With necessaries reduced to a minimum, an easy, middle-aged pair of shoes, and a congenial companion, a walk through Devonshire, North Wales, or the Lake country is by no means difficult, and shows one much more of the beauties of the country than any other sort of locomotion by road or rail. If you think of the neighbourhood of your own home you will remember that half the special places you take your London friends to see are not to be reached by wheels, not even by a bicycle. There is the ferry under the hanging wood where the river bends round through the flat green meadows which stretch up to the foot of the old church half a mile away on its little hill; and the steep grassy lane worn away during the lapse of centuries till the tops of its high hedges are hardly above the level of the fields on each side—these and thousands of other places like them can only be seen by walkers. In this country we care for little tours of this sort less than some Continental nations, Germans, for example. If one sits by any high-road in Switzerland for an hour or two on a summer's day one sees pilgrims with knapsack and alpenstock trudging along to the nearest pass or point of view. Men and women, young and old, they pass by with cheerful, sunburnt faces and, except for an occasional couple of unmistakable young Englishmen, they are all "foreigners." Of course, a walk in England should not be on the high roads, unless in such places as the New Forest, where it might lead to much confusion to attempt short cuts. A map showing lanes and field-paths is indispensable, and a pocket compass desirable; and, armed with these, you will find that a dozen miles a day will cover a considerable amount of very charming country in a week. But it cannot be laid down too firmly that not distance, but enjoyment of the walk is the thing to be aimed at. The cyclist who watches her cyclometer crawling up to beat yesterday's record, instead of taking in the beauties of the road, might almost as well be on a treadmill; and a pedestrian should not feel the day wasted if her walk has been a very short one, as long as it has been crowded with interest. If twelve miles is too much for comfort, walk ten; or do half in the early morning, and then rest in some enticing spot till the shadows grow again in the evening. After skirmishing about crowded stations, rushing to catch trains, and dawdling about platforms waiting for late ones, there is the most delightful sense of freedom in being quite independent of arrangements made by other people.

Again, nurses who live in places where many of them are congregated together find a quiet place a much greater change, and therefore rest, than a noisy seaside town; and those whose work takes them into quiet country districts, and whose evenings are spent in their own society, do well to choose a place where they can see how the world is going on. They will feel a week or two of London quite invigorating, and will go back to their lonely work much cheered up by the refreshing view of shops, theatres, and finery!

But it is certain that all city hospital nurses should spend their holiday time out of doors. It is a good rule for them, when they are choosing lodgings, never to go to a place where it is necessary to put on a hat to sit out of doors. That is, a place should be chosen where there is a garden or a balcony, and a landlady obliging enough to serve at least breakfast and tea *al fresco*. Eight hours of fresh air daily is the least amount to be aimed at, and a good deal more can be secured if the garden boasts of a shady tree where a hammock can be slung, and the hot afternoons spent in a lazy little nap.

### SOME IDEAS ABOUT HOLIDAYS.—*Continued.*

August is for many people the most convenient month for holidays, but it is far from the pleasantest. From long before Chaucer's days, people thought of May as the month for pilgrimages, and the country is much more beautiful in June and the early part of July than in August. Most of the wild flowers are gone by then, and there is a sort of steady unrelieved greenness in the trees and hedges in place of the many-coloured tints of the earlier and later months. In August, trains, lodgings, beaches, steamers, are all crowded; landladies are condescending, and cabs at a premium, while in these places which hold out a scanty welcome in the height of the season there will be affable smiles, and probably lower charges, in June or October.

It is not judicious to take the same sort of holiday year after year. "Elia" has something to say on this subject. "Somehow or other my cousin contrives to wheedle me, once in three or four seasons, to a watering-place. Old attachments cling to her in spite of experience. We have been dull at Worthing one summer, duller at Brighton another, dullest at Eastbourne a third, and are at this moment doing dreary penance at—Hastings! and all because we were happy many years ago at Margate. That was our first seaside experiment, and many circumstances combined to make it the pleasantest holiday of my life." A repetition of the same sort of holiday, with the same companion, is very apt to fall flat. If a fortnight was spent last year in Belgium with a cycling friend, it is better to resist trying Normandy with her this year, but to look for a cottage in some quiet place by the sea, where a party can be made up for a few weeks; or to find a pleasant boarding-house where one may rub off the rust of one's mind by making acquaintance with some nice people.

There can be no doubt that change is such a necessity for a holiday that medical or nursing literature should be laid aside for the time in favour of quite unprofessional reading. It has also been hinted that the inveterate novel-reader would be none the worse for exchanging her romance, and even her "novel with a purpose," for a bit of fancy work or a game of patience! But this is a hard saying to some of us.

Of course, nurses should drop their profession with their uniform as far as possible in their holidays. It is astonishing how, when a nurse has once made known her calling, she is assailed with accounts of sudden seizures, and of fearful and quite impossible operations, if not indeed of "hospital scandals," as it is taken for granted that she is a charmed listener to such tales.

It is sometimes rather an effort to hold one's peace. There were two nurses staying in a little mountain hotel in Switzerland, in strictest mufti, having threatened each other with the direst penalties if the word "Sister" were ever heard from either of them. They kept themselves in check excellently, sat like blocks of wood when any question of nursing or nurses came up, till the very last evening before they left. Then a good lady who spent her life in travelling on the Continent was regretting the difficulty of getting recent books. But she had just read the "Christian," and was much pleased with it, and proceeded to dilate on its excellencies. Could any two nurses resist this? Number one looked at number two, who nodded ready acquiescence, and then they took up their cudgels and plunged into the fray, and Hall Caine's hospital and heroine were once more knocked to pieces, and shown to be only the make-believe that we all know them to be. And the bystanders joined the nurses in their laugh against themselves when they confessed the reason for their vehemence.

But however they were spent, the holidays are over now; the occasional discomforts of them forgotten, the sunny days thankfully remembered, and the old routine begun again for another year. And there is the hope that with another summer will come an equally delightful time, when we can say once more, in the words of the old rhyme:

"Work is over, God will speed it:
Work and workmen with Him rest.
His good blessing, much we need it,
That alone can make us blest.
Rest is come, with joy receive it;
We have done the best we can.
Work is over, here we leave it,
End of God and means of man."

### Everybody's Opinion.

[Correspondence on all subjects is invited, but we cannot in any way be responsible for the opinions expressed by our correspondents. No communication can be entertained if the name and address of the correspondent are not given as a guarantee of good faith, but not necessarily for publication. All correspondents should write on one side of the paper only.]

### THE LATE NURSE OSGODBY.

"Mary Girdlestone," infirmary matron and superintendent of nurses at Manchester Workhouse Infirmary, Crumpsall, writes: I shall be greatly obliged if you will insert this letter in your next issue, as I feel that all Crumpsall nurses, and especially the friends of the late Miss Osgodby (not Asgodby), would like to hear a few more details than those supplied by the account of the inquest last week. I had known Miss Harriett Osgodby for nearly six years; first as a probationer, then as ward sister, and for the last two years as my second assistant matron, or superintendent, as they are called here. She was a most capable and conscientious nurse, particularly clever in surgical work, and very successful in teaching probationers. Miss Osgodby was much beloved by everyone here, and I feel that I have lost a friend as well as a most faithful and efficient helper. She had suffered from a weak heart for some time, and was subject to occasional fits of depression, but had seemed particularly bright and cheerful the day before her death, which occurred on Saturday morning, not evening, as stated. What led to the terrible act which caused it will always remain a mystery. There was nothing to lead us to think that it had been premeditated, and no one who knew her could imagine it possible, except on the hypothesis of temporary insanity. The funeral last Tuesday, when she was laid to rest in the Southern Cemetery, near Manchester, was attended by all the nurses who could be spared from duty, and more than forty of us gathered round her grave. The coffin was covered with beautiful flowers, sent by her friends and by the medical and nursing staffs of the infirmary, who were anxious to show their affection and respect for one whom all sincerely mourn and whose tragic death is deeply deplored.

### THE SCARCITY OF JEWISH NURSES.

"R. B." writes: I was much interested in reading your comments on the scarcity of Jewish nurses. It has been my privilege to nurse a good deal amongst the Jews, both the poor of the East End, and also the better classes. I can say that I have never to my knowledge met one nurse of their own denomination. Surely it is a great pity, because in many instances a well-trained nurse of their own persuasion would be of great value, especially when one considers that the last sacred offices for the dead, for instance, are never performed by Christian hands. In my opinion educated Jewish women would find a vast field of usefulness open to them in the nursing profession, and they would be welcomed in many homes, although the members of the Jewish faith do not object to a Christian nurse. On the contrary, I have always met with gratitude and every consideration from them at all times.

### THE HOME SISTER'S AUTHORITY.

"Nurse Elizabeth" writes: As "Fairplay" has asked my opinion about some of the numerous trials of "A Liverpool Nurse," I give it that, if the home sister sees fit, the sick nurses should be attended to by any nurse she chooses to honour with the duty. Why does "A Liverpool Nurse" object to nursing one of her sick sisters? Is it because it takes a little of her off-duty time? I know by experience that a sick nurse's room could be filled all day with idle, gossiping nurses; but it is really too fatiguing to give the poor girl her morning or evening wash—and very little more is required, as if a nurse is seriously ill she is put in a "sick-room" and attended to by a special nurse. Let us hope for the sick one's sake that "A Liverpool Nurse" will not be told off for that duty, as services grudgingly given would surely retard anyone's recovery. As to the clothes sorting, it requires a brave and happy disposition to perform any humble work cheerfully, and that disposition is not possessed by "A Liverpool Nurse." I am sure "Home Sister" would allow the night nurses to stay longer in bed, and the day nurses to retire earlier, if they had lost any rest through helping her.

"Justice" writes: Will you allow me space to give "A Liverpool Nurse" a little sensible advice? A person so full of grievances awakens my greatest pity! Instead of publishing her "wrongs" let her go straight to the home sister and quietly tell her all she has told you. In case of her getting no satisfaction from the home sister, then let her consult the lady superintendent of the hospital or the committee; that would allow the poor home sister a chance of defending herself, which she cannot do at present, as no nurse with any respect for herself would take the slightest notice of things said at her. She herself was straightforward in her complaints, going to the nurses themselves and saying what she had to say. In these days when nurses are protected by committees there is no excuse for "A Liverpool Nurse's" cowardly act in attacking her home sister through the Press.

### Appointments.

Chelsea Infirmary.—Miss Annie Garson, Miss Mary Roberts, and Miss Edith Hancock have been appointed sisters. Miss Garson and Miss Roberts were trained at Chelsea Infirmary. Miss Roberts was trained at the Royal Hants County Hospital, where she has since been ward sister. Miss Lilian Ainsworth has also been reappointed sister on her return from South Africa, after serving for nearly three years in the Army Nursing Reserve.

City Fever Hospital East, Old Swan, Liverpool.—Miss Jeannie Booth has been appointed night sister, Miss Catherine A. Elwood and Miss Clara Towers sisters. Miss Booth and Miss Towers were trained at the Royal Infirmary, Preston; Miss Elwood at the Toxteth Infirmary, Liverpool.

Royal Devon and Exeter Hospital.—Miss Emma Smale has been appointed matron. She was trained at the Eastern Fever Hospital, London, and at the Royal Devon and Exeter Hospital, where she has since been successively sister, night superintendent, and assistant matron.

St. Olave's Union, S.E.—Miss F. E. Capes has been appointed matron and superintendent of the Girls' Department at St. Olave's Union Temporary School, Sutton. She was trained at St. George's Infirmary, and was afterwards charge nurse at Sutton Schools for six years, charge nurse at St. Olave's Infirmary for three and a half years, and assistant matron at St. Olave's Infirmary, Ladywell, since the opening. Miss J. E. Street has been appointed assistant matron at St. Olave's, Ladywell. She was trained at St. George's Infirmary, where she remained as staff nurse for six years. Subsequently she was for nine years charge nurse at St. Olave's Infirmary, and for one year superintendent nurse at St. Olave's, Ladywell.

### To Nurses.

We invite contributions from any of our readers, and shall be glad to pay for "Notes on News from the Nursing World," or for articles describing nursing experiences, or dealing with any nursing question from an original point of view. The minimum payment for contributions is 5s., but we welcome interesting contributions of a column, or a page, in length. It may be added that notices of appointments, entertainments, presentations, and deaths are not paid for, but that we are always glad to receive them. All rejected manuscripts are returned in due course, and all payments for manuscripts used are made as early as possible after the beginning of each quarter.

### For Reading to the Sick.

SUNDAY.

On this day, the first of days,
God the Father's name we praise;
Who, creation's Lord and Spring,
Did the world from darkness bring.

On this day the Eternal Son
Over death His triumph won;
On this day the Spirit came
With His gifts of living flame.

O that fervent love to-day
May in every heart have sway,
Teaching us to praise aright
God the Source of life and light.

Father, who didst fashion me
Image of Thyself to be,
Fill me with Thy love Divine,
Let my every thought be Thine.

*Sir H. Baker.*

The observance of Sunday is a practical acknowledgment of Almighty God, and of His sovereign claims upon His people. It serves also as a perpetual reminder that all our time belongs to God, and that we are bound "to serve Him truly all the days of our life." Just as devout Christians set aside a fixed proportion of their income for God's service, as an acknowledgment that all their possessions are the Lord's; so it is in this matter of setting apart one day in every seven for His special service.

Certainly the whole life of a Christian should be a consecrated life: God is not to be forgotten in the week because He is especially remembered on Sunday. All a Christian's time is properly consecrated time; but practically, in many cases, none would be consecrated unless an effort were made to mark a certain proportion of it by a special consecration. The case is parallel to that of prayer. Our Lord says that "men ought always to pray, and not to faint" (St. Luke xviii. 1). The apostle says, "Pray without ceasing" (1 Thess. v. 17). And the life of a good Christian is, no doubt, a continuous prayer; the spirit of prayer penetrates and hallows it; each duty is intertwined with acts of the soul which raise it above this earthly scene to the throne and presence of Christ. But, for all that, in all Christian lives stated times of prayer, private as well as public, are practically necessary, if the practice of prayer is to be consistently maintained. Yet morning and evening and mid-day or other devotions are perfectly consistent with recognition of the apostolic and divine sayings, that prayer should be incessant in a Christian life. It implies that all our time belongs to God, although, considering our weakness, He graciously accepts a prescribed instalment or section of it.—*Liddon.*

When I have said my quiet say,
When I have sung my little song,
How sweetly, sweetly dies the day
The valley and the hill along;
How sweet the Summons "Come away"
That calls me from the busy throng!
I thought beside the water's flow
Awhile to lie beneath the leaves;
I thought in Autumn's harvest glow
To rest my head upon the sheaves;
But, lo! methinks the day was brief
And cloudy; flower, nor fruit, nor leaf
I bring—and yet accepted, free,
And blest, my Lord, I come to Thee.

*D. Greenwell.*

### A Book and its Story.

THE LATE MRS. JAMES STUART-WORTLEY.

In these short biographies of twelve "Noble Women of Our Time" the author brings before the reader the names of those whose lives were spent in the service of their Master, and who passed to their rest after years of suffering which was not allowed to sever their connection with, nor abate their interest in, the good works with which their names will be for ever associated. He considers that full justice has yet to be done to the memory of the many heroic women who, if they have not made history, have yet by their influence had a real and lasting effect on the most important movements of their day. He considers also that this is due indirectly to the fact that as history has been written by men, so they fail to delineate adequately, from want of capacity to fathom it, the true character of the best and wisest of the sex. He opens his little biographies with a tribute to the complex and, to the average man, mysterious nature of the true woman. "Were it possible to look into the heart of nine men out of ten, there would be found a love and wondering admiration for the good women they have known. For a man can hardly ever understand the depth of devotion, the rapid conviction, the manifold motives of a woman . . . and many a man could bear witness to the debt that can never be paid—that he and all men owe to the devotion and wiseheartedness of women."

Among those lives selected by Mr. Douglas How for special notice, that of the Hon. Mrs. James Stuart-Wortley should have interest for Hospital readers as her name is associated with the first movement towards providing nurses for attendance on the sick poor in their own homes. "In this special movement she was almost a pioneer. Out of her own pocket she provided a nurse for the parish of Christ Church, Watney Street, and continued the payments up to the day of her death, although there must have been times when the money could with difficulty be spared." As an outcome of the experience gained in the terrible cholera epidemic of 1896, the East London Nursing Association was formed in 1898. From the outset Mrs. Stuart-Wortley interested herself greatly in its progress, and later she became treasurer to the Association, and was instrumental in prevailing upon Princess Christian to become its President; she also assisted greatly in raising funds by meetings at Grosvenor House and Lowther Lodge in support of the Society. She had had much personal experience in the sick room, for her husband had a long and trying illness in which she was his devoted nurse. "She delighted in *personal* service. Her personal and practical sympathy and help were never failing and it is said by one who knew her well, 'that the combination of intellect and sympathy was almost more than I could have thought possible.' Mrs. Stuart-Wortley was also a very beautiful woman; her visits to the sick poor were veritable angel visits, and were, we are told, a source of untold happiness and renewed cheerfulness and hope to them. But it was not only the sick to whom her kindly attention was given. The nurses who were working among the poor of East London owed much to her for the thoughtful sympathy and care which she showed to them. Her advice was to take as much care of themselves as of their patients. Neglect of this rule she considered was equal to neglect of their patients. And many weary overworked matrons of the association can look back gratefully to the few days of rest at her house, "where all was so beautiful, so simple, and so refined." A story is told which shows that the sweetness of Mrs. Stuart-Wortley's character was toned with an undercurrent of sternness which was in evidence when necessary. "A very advanced 'modern' lady once asserted herself strongly in conference with doctors and other gentlemen. 'My dear Miss ——,' said Mrs. Stuart-Wortley, 'you have been glad that I should introduce you to these leading members of professions and of society, therefore you must let me say that when you are with gentlemen you must not behave to them as one gentleman dare not behave to another.'" Until the close of her life she continued to take the liveliest interest in the work of the nursing profession, and in one of the speeches which she made on behalf of the East London Nursing Association occur the following words, "I am an old woman, but I should like before I die to see all London covered with an effective nursing organisation." Among many other philanthropic works in which Mrs. Stuart-Wortley interested herself was that of the British Women's Emigration Society, and in this work the "Imperialism" which so strongly characterised her was a strong factor. "She kept a perpetual watch over Colonial matters, and assured herself that the interests of both emigrants and Colonies were properly studied. . . . Her personality," the writer continues, "and her magnificent breadth of character, made her a woman whom to know was to love and endeavour to follow."

The name of Mrs. Sidney Lear is one too well known to render it necessary to remind our readers that she is the writer and compiler of some of the most helpful devotional books in the language. "The Light of the Conscience," "Life of Fénelon," "The Devout Life," "A Dominican Artist," and many others are from her pen. Written with a breadth of view and in scholarly and cultured literary style that suggests the student—although the delicate sympathy and tact that characterises all she has written are eminently feminine—the signature of "Sidney Lear," may have left the sex of the writer to many readers an open question. Without doubt the whole training of her life tended to make her literary work helpful to those distressed in mind or body, for we read, "The whole disposition of it seems to have been so ordered by Providence that her chief care should be for others." A large number of her best years were spent in nursing. "When no longer young she married the Rev. Sidney Lear, brother-in-law and chaplain to Bishop Hamilton of Salisbury. Mr. Lear, never strong, was practically kept alive for the few years of their married life by her unremitting care." After the death of her husband her literary work was suspended for a time to be resumed later, and the years of waiting were not wasted ones. The books written by Mrs. Lear during her widowhood show a deepened spirituality for which these years were a training and preparation. No one has written more brightly and sensibly on the duty of cheerfulness and the cultivation of it as a necessary condition to physical and mental well-being. And, as might not be expected, we learn that her advice was the outcome of her own inner experience of times of the deepest depression. Hers was indeed a wonderful life and a wonderful character, when her vast literary work is considered as well as the large and important charitable enterprises in which she was engaged. Early in November 1896 the end came after eight years of failing sight and health. She was buried beside her husband in the cloisters at Salisbury, and of the crowd who gathered around her grave numbers belonged to the working classes, bearing witness to one of the ruling passions of her life—love for the souls of men.

"Noble Women of Our Time." By F. Douglas How. (1 vol. Price 6s. Published by Isbister & Co.)

### Echoes from the Outside World.

### Movements of Royalty.

THE Braemar gathering last week—which took place at Cluny, in a large field close to the banks of the Dee—was very extensively attended, and the large number of visitors recalled the occasion of Queen Victoria's Jubilee, when the gathering was held at Balmoral. There was no meeting last year owing to the late Queen's death, and the year before it was postponed because of the war in South Africa. The King and Queen, the Prince of Wales, Princess Victoria, and the Duke and Duchess of Fife were present, and a special pavilion had been erected for their use by Mr. Farquharson of Invercauld, upon whose ground the festivity was held. The Balmoral party arrived about half-past three, and it was at once observed that little Prince Edward was with his grandfather, whilst Prince Albert of Wales accompanied his father in another carriage. All four wore Highland costume, the kilts being of the Balmoral tartan. Upon the arrival of the Royal visitors, the Highlanders mustered for a march round the field accompanied by their respective pipers. Three times the clansmen made the tour of the ground, and each time the enthusiasm of the onlookers increased in fervour. The Queen took photographs of the proceedings. Later there were contests in dancing, leaping, vaulting, hammer-throwing, tossing the caber, etc. The hurdle-race, in which the competitors had to go through the River Dee, evidently much amused His Majesty. The King and Queen remained more than a couple of hours, and the weather was pleasant the whole time. On Tuesday the Queen and Princess Victoria visited Lord Rosebery at Dalmeny Park, on their way to Denmark. It is announced that the King and Queen will go into the City of London on Saturday, October 18, returning to Buckingham Palace by way of certain of the South London thoroughfares. The next day they will attend a Thanksgiving Service at Westminster Abbey. No details are as yet officially announced.

### The Czar and His People.

THE Czar has been making short but significant speeches at Kursk. On Saturday, on his way to the manœuvres, His Majesty, who was received at the railway station by the representatives of the nobility of the province and of the provincial Zemstvo, after thanking the former for their welcome, said, "The affairs of the landed nobility are going through a severe crisis, and those of the peasants are to a certain extent in disorder. By my orders the measures to meet these difficulties are being studied at the Ministry of the Interior. Provincial committees in which the nobility and the Zemstvo will participate will be called upon in good time to co-operate in this work. The system of landed estates has for ages been the mainstay of order and morality in Russia, and its maintenance will be an object of my constant solicitude." The Czar, addressing the Zemstvo representatives, said that he should be happy to give them every protection, "while at the same time taking steps to secure the unification of the various local authorities." Subsequently, he received deputations of the elders of the cantons and villages in the house of the Governor of Kursk, and having alluded to the plunder of certain estates by the peasants in the spring, he continued: "Remember that a man gets rich, not by seizing the property of others, but by honest labour and thrift, and by living according to the commandments of God."

### Profound Peace in Cape Colony.

IN the Cape House of Assembly on Monday the Bill indemnifying the Government and other officials of Cape Colony in respect of the violation of certain laws in connection with the framing of voters' rolls was read a first time. On Tuesday it was announced by Sir Gordon Sprigg that martial law had been abolished, and that the Peace Preservation Act, which enables the authorities to control the possession, importation, and registration of all arms and ammunition, had been put into force. According to the Attorney-General's statement, profound peace now exists throughout the Colony.

### Opening of the Dutch Parliament.

ON Tuesday Queen Wilhelmina, who received an enthusiastic welcome on the occasion of her first public appearance at The Hague the previous day, opened the session of the States-General. She was accompanied to Parliament by Prince Henry and the Queen-Mother, and looked remarkably well. Among the Bills announced in the speech from the Throne were measures for extending facilities for intermediate and higher education, for developing technical education, for restricting compulsory vaccination, and for making better provision for granting pensions to teachers in private schools and the widows of teachers in general. It is also intended to introduce Bills for amending the labour law, for regulating lotteries, and for abolishing the State lottery.

### The Boer Generals.

MR. CHAMBERLAIN, by request, accorded lately to the Boer Generals an interview at the Colonial Office, and a Blue-book on the subject was issued last week. The Generals had previously intimated that they had eleven specific proposals to make, and that there were other points which they wished to submit for consideration. The Colonial Secretary at once replied that the number and character of the demands had greatly surprised him, and that it was the intention of his Majesty's Government to observe the terms arranged at Vereeniging, both in the spirit and in the letter, but he could not now receive fresh proposals, nor could he enter upon any discussion on the matter. Thereupon the Generals gave a formal assurance not to raise any subject inconsistent with the terms of surrender and the interview was granted. At the close Mr. Chamberlain made a statement to the effect that this country wanted to forget and forgive, and that now there was peace all the British wished was to recognise the Boers as fellow-subjects working with them for the prosperity and liberty of South Africa. The promise of the English Government to pay the £3,000,000 as compensation for Boer losses and damages out of the British exchequer, not from that of the new colonies, is looked upon in Boer circles very hopefully, and as a direct consequence of the entreaties of the Generals. £2,000,000 will also be paid as compensation to British subjects who have suffered loss during the late war.

### Poison in the Pork-Pie.

A number of persons have been poisoned from eating pork-pie purchased at Derby, and three, including a husband and wife residing at Taunton, have died. On returning from Derby, where the latter bought the pie, they partook of it at supper, and again at breakfast on the following morning. At a meeting of the Public Health Committee of the Derby Corporation on Monday the Medical Officer submitted the report of Professor Delepine, who, having analysed one of the incriminated pork-pies, found that either the meat or the jelly, or both, used in the pies became infected in the course of preparation. He states that he has been able to isolate a bacillus belonging to the colon group, which he declares is undoubtedly responsible for the pathogenic properties acquired by the pork-pies. Negligence is not imputed to the vendor, but pork-pies in Derby are for the time a drug on the market.

### Notes and Queries.

**The Editor is always willing to answer in this column, without any fee, all reasonable questions, as soon as possible.**

**But the following rules must be carefully observed:—**

**1. Every communication must be accompanied by the name and address of the writer.**

**2. The question must always bear upon nursing, directly or indirectly.**

**If an answer is required by letter a fee of half-a-crown must be enclosed with the note containing the inquiry, and we cannot undertake to forward letters addressed to correspondents making inquiries. It is therefore requested that our readers will not enclose either a stamp or a stamped envelope.**

*Milk.*

(149) I should be so much obliged if you can tell me why peptonised milk curdles sometimes when I boil it. I use Fairchild's zymine peptonising tubes and follow directions; sometimes the milk is all right and sometimes it curdles.—*Nurse W.*

The under peptonisation may be due to many causes:—(a) The failure to follow directions in every detail, *e.g.*, the milk and water too cold at the time that the contents of the tube are added; (b) Preservatives in the milk restraining the action of the ferment on the casein; (c) Acidity of the milk due to it having been kept too long; (d) An exceptionally high percentage of casein. The last, however, is a rather rare occurrence: at this season of the year the presence of increased quantities of preservatives or acidity of the milk are more likely to be the causes of the curdling.

*State Registration.*

(150) How can I join the Society for the State Registration of Nurses, and to whom should I apply?—*Matron.*

Address the Secretary, the Matrons' Council of Great Britain and Ireland, 46 York Street, Portman Square.

*Post-Graduate Training.*

(151) I should be much obliged if you can give me the addresses of any hospitals abroad where nurses can obtain a post-graduate diploma?—*Nurse May.*

You can obtain post-graduate training in some of the American hospitals. See reply to *M. M. G.*

Is there any hospital in London where nurses can receive a post-graduate training for about three months as in some of the American hospitals?—*M. M. G.*

We have not heard of special arrangements being made for post-graduate hospitals in England, but doubtless private arrangements could be made with those institutions which receive paying probationers.

*Unemployed.*

(152) Most of the working classes have societies to help them when they are out of work, but I see there is nothing for nurses. Last October I had a case of typhoid, and contracted the disease. It was fourteen weeks before I could do anything, and then I have only had seven weeks' work. I have now been at home nearly three months, and I cannot get work through no fault of my own. What can I do?—*Nurse J.*

You should belong to the Royal Pension Fund for Nurses, and to the sick fund belonging to that society, so that when you are so unfortunate as to have a long illness you will receive sick pay as do the working classes. There are plenty of nurses wanted in Poor Law institutions. Apply for vacancies, for which see advertisements.

*Advice.*

(153) I am 26 years of age, and was trained as parish sister by the Women's Association of Home Missions, Church of Scotland. An accident to my ankle has left me slightly lame, and this has obliged me to give up my post as parish sister, and also one as sub-matron in an asylum for imbecile children. My matron advises me to receive mental patients in my own home, as my mother has also had experience in mental nursing. Any suggestion that you can kindly make will be of great service.—*M. J. C.*

If you can get private patients in your own home, do so; but if you take mental cases, you must write to the Clerk of the Lunacy Board for Scotland, 51 Queen Street, Edinburgh, and obtain particulars about registering your home for the purpose.

*Address.*

(154) I should be glad if you will tell me if there is an "All Souls' Nursing Guild," and if so, give me the address of the Superior.—*M. E. J.*

We have not the address, but perhaps one of our readers can kindly supply it.

*Phthisis.*

(155) Can you kindly tell me of a home where a girl suffering from consumption can be received for the sum of 6s. or 8s. a week. Her parents are poor and cannot afford to pay for her.—*M. A. L. P.*

Apply to the Mount Vernon Hospital for Consumption, Hampstead, N.W. Also to the Principal of the Winter Home for Consumptive Girls, Ribbsford House, Chapel Park Road, St. Leonards.

A young nurse, after having worked for a time in a consumption hospital has since developed the disease. She has been 14 weeks at the Royal National Hospital, Ventnor, but is not yet cured. Is there a cheap home where she could be admitted? Could you give me a list of open-air sanatoria?—*C. M. V.*

You will find a short list of open air sanatoria in "Burdett's Hospitals and Charities." A nurse might possibly, by an advertisement, find a suitable home in one of the many private sanatoria at advantageous terms. The Maitland House Sanatorium, Kidmore, Reading, might do so.

Can you kindly tell me where I could send a young man suffering from consumption for open-air treatment. He could afford to pay a guinea a week, but not more.—*M. R. H. P.*

The Richmond House Sanatorium, Clare, Suffolk, might be suitable perhaps.

*Colonial Nursing Association.*

(156) I should be much obliged if you will kindly tell me the address of the Colonial Nursing Association.—*L. H. and Miss W.*

The Imperial Institute, S.W.

*Home.*

(157) Will you kindly tell me of any convalescent homes for ladies at Rhyl, Aberystwith, or other places on the coast of Wales where a nurse in delicate health could be received by paying a small sum weekly?—*An Inquirer.*

You might be received perhaps at the Royal West of England Sanatorium, Weston-super-Mare.

Can you recommend me an institution where a lady suffering from melancholia with suicidal tendencies could be received on easy terms?—*E. F. V.*

The case seems most suitable for a special hospital such as the Bethlem Royal Hospital, Lambeth Road, S E.

*Weaning.*

(158) Would any nurse tell me the best manner to wean a baby. He is six months old and a very big vigorous boy, but at each attempt to wean him he has become ill. He can have milk fresh from the cow.—*Inquirer.*

The subject is too large to enter upon here. You might consult "Our Baby," by Mrs. Langton Hewn (John Wright and Co.), or "The Physiological Feeding of Infants," by Dr. Eric Pritchard (Scientific Press).

*Wine.*

(159) Can you recommend any wine that is good for gouty people with bad digestions?—*Muriel.*

You had better consult your doctor. As a rule wine is not recommended to gouty patients.

*Child.*

(160) Will you kindly tell me if it is harmful for a child of 11 months to hold on to things and to stand? Its mother is anxious, as she thinks it has a tendency to bow-legs. When ought a child to give up having the bottle before going to sleep?—*E. M. H.*

Very much depends on the child. Many babies are walking at 12 months of age. Use discretion both in allowing the child to walk, and weaning it from its bottle. If the child has been bottle-fed, and has a tendency to bow-legs, the chances are that it has rickets. Consult a doctor.

*Help.*

(161) Would you kindly let me know if there is any charity from which a sick nurse could receive a little help? She is not a member of the National Pension Fund—*W. W.*

Apply to the Hon. Secretary of "The Hospital Convalescent Fund," care of the Editor of The Hospital.

**Useful Handbooks for Nurses.**

"Nurses' Dictionary of Medical Terms." Cloth, 2s.; leather, 2s. 6d.; post free 2s. 8d.

"On Preparation for Operation in Private Houses." 6d.

"Hospital Sisters and their Duties." 2s. 6d.

"Medical Gymnastics, including the Schott (Nauheim) Movements." 2s. 6d.

"The Human Body." 5s.

"Practical Handbook of Midwifery." 6s.

"A Handbook for Nurses." (Illustrated.) 5s.

"Tendencies to Consumption: How to Counteract Them." 2s. 6d.

"Syllabus of Lectures to Nurses." 1s.

The above works are published by the Scientific Press, Ltd., and may be obtained through any bookseller or direct from the publisher, 28 and 29 Southampton Street, Strand, London, W.C.

## Figures and Tables

**Fig. 59. Fig. 60. f1:**